# PPAR Genomics and Pharmacogenomics: Implications for Cardiovascular Disease

**DOI:** 10.1155/2008/374549

**Published:** 2008-03-24

**Authors:** Sharon Cresci

**Affiliations:** Department of Medicine, Washington University School of Medicine, Washington University, Saint Louis, MO 63110, USA

## Abstract

The peroxisome proliferator-activated receptors (PPARs) consist of three related transcription factors that serve to regulate a number of cellular processes that are central to cardiovascular health and disease. Numerous pharmacologic studies have assessed the effects of specific PPAR agonists in clinical trials and have provided insight into the clinical effects of these genes while genetic studies have demonstrated clinical associations between PPAR polymorphisms and abnormal cardiovascular phenotypes. With the abundance of data available from these studies as a background, PPAR pharmacogenetics has become a promising and rapidly advancing field. This review focuses on summarizing the current state of understanding of PPAR genetics and pharmacogenetics and the important implications for the individualization of therapy for patients with cardiovascular diseases.

## 1. INTRODUCTION

PPAR-alpha (PPAR*α*), PPAR-beta/delta (PPAR*β*/*δ*), and PPAR-gamma (PPAR*γ*) are nuclear hormone receptor transcription factor proteins encoded by similarly named genes (*PPARA*; *PPARD*; *PPARG*) [1, 2]. Each of the PPARs has multiple promoters and more than one isoform, resulting from alternate splicing, alternative transcription start sites or both [3–5]. The PPARs have distinct, but overlapping, tissue expression patterns and act to coordinately regulate multiple metabolic pathways [1, 2].

PPAR*α* is highly expressed in the heart, liver, and skeletal muscles [2]. In these tissues, PPAR*α* is the central regulator of genes involved in fatty acid metabolism and appears to mediate the balance between cellular fatty acid and glucose metabolism, particularly at times of metabolic or physiologic stress, such as myocardial ischemia, hypertrophy, heart failure, and insulin resistance [6–15]. In addition, PPAR*α* is involved in the energy substrate and fiber-type switches that occur in skeletal muscle as a result of conditioning [16] and is involved in the inflammatory response during vascular atherosclerosis [17–19].

PPAR*γ* is highly expressed in both white and brown adipocytes [2, 20, 21]. PPAR*γ* controls adipocyte lipid storage and release and is an important mediator of insulin sensitivity [22, 23]. In addition, PPAR*γ* regulates adipocyte release of adipokines including tumor necrosis factor alpha (TNF*α*), angiotensinogen (AGT), interleukin-6 (IL-6), and plasminogen activator inhibitor type 1 (PAI-1) [24].

PPAR*β*/*δ*, also known as nuclear hormone receptor 1 (NUC 1) or fatty acid-activated receptor (FAAR), is ubiquitously expressed but is expressed at higher levels in the brain, adipose tissue, and skin [2, 25]. PPAR*β*/*δ* is thought to be critically important in adipocyte and skeletal muscle fatty acid oxidation and is another important mediator of insulin sensitivity [26–28]. PPAR*β*/*δ* appears to also be involved in obesity [26–28] and in preventing myocardial hypertrophy via NF-*κ*B inhibition [29–31].

The PPARs are able to bind many different ligands including metabolic intermediates (fatty acids), pharmacologic agents (fibrates, thiazolidinediones), and natural herbs (green tea) [32–36]. In the presence of ligand, PPARs bind to their cognate regulatory elements as a heterodimer with retinoid X receptor *α* [37]. Ligand binding causes a conformational change that results in the recruitment of coactivators and increased transcriptional activation of target genes [34, 35, 38, 39].

There is considerable clinical association data linking polymorphisms of *PPARA*, *PPARD*, and *PPARG* with cardiovascular disease (coronary and carotid atherosclerosis, left ventricular hypertrophy) and cardiovascular risk factors (incidence of type 2 diabetes mellitus (DM), obesity, insulin resistance, and abnormal lipid profiles) in populations of diverse ethnicity. There is less data on PPAR pharmacogenetics, but the field is rapidly growing and of considerable interest to many investigators. PPAR pharmacogenetics of fibrates (gemfibrozil, fenofibrate, and bezafibrate), thiazolidinediones or glitazones (troglitazone, pioglitazone, and rosiglitazone), statins, and acarbose have particular relevance to cardiovascular disease.

This review will discuss several significant PPAR genetic and pharmacogenetic associations that have been observed with respect to cardiovascular disease (Table 1 provides the rs number for each SNP discussed in this review). Understanding the current state of PPAR genetics and pharmacogenetics may have important implications for the future individualization of therapy for patients with cardiovascular disease.

## 2. PPARA

### 2.1. PPARA Leu162Val genetic associations

#### 2.1.1. Dyslipidemias

*PPARA* Leu162Val is a polymorphism located in the DNA binding region of PPAR*α* that confers differential ligand-mediated activation of PPAR*α* in vitro [40, 41]. Investigators from several clinical studies have observed that carriers of the *PPARA* Val162 allele, compared to *PPARA* Leu162 homozygotes, have significantly higher concentrations of serum triglycerides, total cholesterol, LDL cholesterol, and apolipoprotein (apo) B and apoC-III. However, there have been exceptions, and not all studies have found an association with all five serum lipids [41–45]. The larger trial findings, as well as the studies that have negative findings, will be discussed here.

Recently, the association of the *PPARA* Leu162Val polymorphism with serum lipid levels was investigated in 5799 individuals from the Inter99 cohort, a Danish cohort targeted for identifying parameters affecting participation in a diet and exercise intervention in the general population [46]. In this cohort, individuals homozygous for the *PPARA* Val162 allele, compared to PPARA Leu162 allele carriers, demonstrated a 70% greater mean fasting serum triglyceride level (2.2 mmol/L (195 mg/dL) versus 1.3 mmol/L (115 mg/dL), resp.; *P* = .007) and a greater mean fasting serum total cholesterol levels (6.2 mmol/L (240 mg/dL) versus 5.5 mmol/L (213 mg/dL), resp.; *P* = .01) [45].

These findings confirmed previous observations in 2373 participants of the Framingham Offspring Study. When the association of the *PPARA* Leu162Val polymorphism with variation in lipid levels was investigated in these subjects, *PPARA* Val162 carriers, compared to *PPARA* Leu162 homozygotes, had significantly increased serum concentrations of total cholesterol in men (*P* = .0012), LDL cholesterol in men (*P* = .0004), apoC-III in men (*P* = .009), and apoB in men and women (*P* = .009 and .03, resp.) [44]. These same investigators went on to demonstrate that the association of the *PPARA* Leu 162Val polymorphism on plasma triglycerides and apoC-III concentrations was more complex and depended on the person's regular dietary polyunsaturated fatty acid intake. PPARA Val162 allele carriers that had a low polyunsaturated fatty acid intake (<6% of calories) had greater serum triglyceride and apoC-III concentrations,compared to PPARA Leu162 homozygotes, whereas PPARA Val162 allele carriers that had a high polyunsaturated fatty acid intake had lower triglyceride and apoC-III concentrations, compared to *PPARA* Leu162 homozygotes [47].

Other studies have also investigated the association of the *PPARA* Leu162Val polymorphism with serum lipid response to diets of different fat composition. Tanaka et al. studied 59 healthy male students fed a single high-fat meal (60% calories as fat (63% saturated fatty acids, 33% monounsaturated fatty acids, and 4% polyunsaturated fatty acids); 15% calories as protein; and 25% calories as carbohydrate) following a 12-hour fast [48]. *PPARA* Val162 allele carriers had significantly higher fasting (baseline) total cholesterol, LDL cholesterol, and apoB levels, compared to Leu162 homozygotes and this variation in serum lipids was maintained after the high-fat meal [48]. No significant association of the *PPARA* Leu162Val polymorphism with serum triglyceride concentrations (either fasting or postprandial) was observed (apoCIII was not measured) [48]. Paradis et al. investigated the association of the *PPARA* Leu162Val polymorphism with serum lipid response in ten PPARA Val162 allele carriers and ten age and body mass index-matched PPARA Leu162 homozygotes subjected to a high polyunsaturated fat followed by a low polyunsaturated fat diet [49]. At baseline, the *PPARA* Leu162Val polymorphism was not associated with variation in serum lipids [49]. After the high polyunsaturated fat diet, *PPARA* Val162 allele carriers had a significant decrease in plasma apoA-I levels, total cholesterol, and LDL cholesterol (small particles), compared to the *PPARA* Leu162 homozygotes (who demonstrated an increase in plasma apoA-I levels, total cholesterol, and LDL cholesterol (small particles): *P* = .02, *P* = .07 and *P* = .08, resp.) [49].

In contrast to the aforementioned studies, when the association of the *PPARA* Leu162Val polymorphism with variations in serum lipids was investigated in 3012 healthy middle-aged men in the second Northwick Park Health Study (NPHS2, Northwick, UK), no association of the *PPARA* Leu162Val polymorphism with serum lipids at baseline, or in response to therapy, was found [50]. Although it was a smaller study, the Lopid Coronary Angiography Trial (LOCAT), a clinical trial of 395 postcoronary bypass men, with an HDL cholesterol ≤1.1 mmol/L and LDL cholesterol ≤4.5 mmol/L that investigated the progression of coronary atherosclerosis in response to lipid lowering therapy with gemfibrozil, [51, 52] also found no association between the *PPARA* Leu162Val polymorphism and serum lipids either at baseline, or in response to therapy [50].

#### 2.1.2. Coronary atherosclerosis

As discussed above, LOCAT found no association of the *PPARA* Leu162Val polymorphism with variations in serum lipids [50]. However, this study did observe that carriers of the *PPARA* Val162 allele showed significantly less progression of atherosclerosis in both gemfibrozil-treated and untreated groups [50]. No pharmacogenetic (i.e., treatment by genotype) interaction was found [50].

### 2.2. PPARA Leu162Val pharmacogenetic associations

#### 2.2.1. Response to gemfibrozil

The Helsinki Heart Study (Helsinki, Finland) was a primary prevention trial that demonstrated that randomization to treatment with gemfibrozil resulted in a 34% reduction in cumulative cardiac events and a 26% reduction in cardiac mortality [53, 54]. Subgroup analysis demonstrated that overweight men with body mass index between 27–40 kg/m^2^ had the largest reduction in cardiac events in response to gemfibrozil in the Helsinki Heart study [55]. Given that the greatest response to gemfibrozil was observed in this group, the association between genetic variation in the *PPARA* Leu162Val polymorphism and the response to gemfibrozil was investigated in 63 abdominally obese men in a randomized placebo-controlled trial [56]. After 6 months of treatment, carriers of the *PPARA* Val162 allele demonstrated a 50% increase in HDL_2_ cholesterol compared to *PPARA* Leu162 allele homozygotes who only had a 5.5% increase (*P* = .03) [56]. The *PPARA* Leu162Val was responsible for 7% of the variance of the change in HDL_2_ cholesterol and there was a significant genotype-by-treatment interaction between the *PPARA* Leu162Val polymorphism and the increase in HDL_2_ cholesterol [56].

The Veterans Affairs High-Density Lipoprotein Intervention Trial (VA-HIT) study of patients with known ischemic heart disease, selected for low levels of HDL cholesterol (mean of 32 mg/dL), demonstrated that randomization to gemfibrozil therapy resulted in a 22% reduction in relative risk of coronary events and a 31% reduction in cerebral vascular events [57–59]. In VA-HIT, the subgroup that benefited the most in reduction of cardiovascular events in response to gemfibrozil were those patients that had DM or insulin resistance [60, 61]. Given that this group had demonstrated the greatest response, the association between genetic variation in the *PPARA* Leu162Val polymorphism and the response to gemfibrozil was investigated [62]. VA-HIT patients with DM or insulin resistance treated with gemfibrozil who were *PPARA* Leu162 homozygotes had a greater absolute reduction in cardiovascular events (12.1% reduction compared to treatment with placebo; *P* = .06) compared to carriers of the *PPARA* Val162 allele who had a nonsignificant reduction (9.9% compared to treatment with placebo; *P* = .28) [62]. Furthermore, in VA-HIT patients without DM or insulin resistance, carriers of the Val162 allele had a significant increase in cardiovascular events in response to gemfibrozil (7% increase compared to treatment with placebo; *P* = .01) [62].

#### 2.2.2. Response to fenofibrate

The Genetics of Lipid Lowering Drugs and Diet Network (GOLDN) investigated the response to fenofibrate (160 mg) for ≥21 days in 791 men and women enrolled in The Family Heart Study (FHS, a multicenter, family pedigree study aimed to identify genetic and environmental risk factors of cardiovascular disease) [63]. Overall, there was a 37 mg/dL reduction in fasting serum triglyceride levels after treatment with fenofibrate (the average of two separate measurements obtained prior to treatment and at the end of treatment were used). Although only reported in abstract form to date, variation in *PPARA* Leu162Val polymorphism was significantly associated with fasting triglyceride level response to fenofibrate treatment [64]. Individuals homozygous for the *PPARA* Val162 allele had a 73 mg/dL reduction in their fasting triglyceride response to fenofibrate compared to *PPARA* Leu162Val heterozygotes (46 mg/dL reduction) and *PPARA* 162Leu homozygotes (53 mg/dL reduction; *P* < .0001) [64].

### 2.3. PPARA Val227Ala genetic associations

#### 2.3.1. Dyslipidemias

*PPARA* Val227Ala is a polymorphism located between the DNA binding and ligand binding domain of PPAR*α*. This region is thought to be important in heterodimerization but in vitro experiments confirming a functional difference in alleles have not yet been performed [65]. The association of the *PPARA* Val227Ala polymorphism with serum lipid levels was investigated in a study of 401 healthy Japanese individuals presenting to medical clinic for routine health care [65]. After adjustment for age and body mass index, female carriers of the Val227 allele had significantly lower serum total cholesterol (*P* = .046) and triglyceride levels (*P* = .038) compared to Ala227 homozygotes [65]. Male carriers of the Val227 allele also had lower serum total cholesterol and triglyceride levels compared to Ala227 homozygotes, but the differences were not significant (*P* = .30 and .54, resp.) [65].

Recently, the finding of this small study was confirmed in 2899 Chinese individuals from the 1998 Singapore National Health Survey (NHS98) [66]. Women *PPARA* Ala227 allele carriers had significantly lower serum total cholesterol (*P* = .047) and triglyceride levels (*P* = .048), compared to *PPARA* Val227 homozygotes, and men had lower levels that were, again, not significant (*P* = .65 and .12, resp.) [66]. In addition to these findings, this study also found a significant interaction between the *PPARA* Val227Ala polymorphism and serum HDL cholesterol levels in response to dietary polyunsaturated fatty acid intake in women suggesting a gene-environment interaction (*P*-value for interaction = .049) [66]. Specifically, the authors found that, in women who were *PPARA* Ala227 allele carriers, increasing dietary polyunsaturated fatty acid intake resulted in lower serum HDL cholesterol levels. This result was in contrast to male *PPARA* Ala227 allele carriers, who had an increase in serum HDL cholesterol levels, and women who were *PPARA* Val227 homozygotes, who demonstrated less lowering [66].

### 2.4. PPARA IVS7 2498 G > C genetic associations

#### 2.4.1. Coronary atherosclerosis

*PPARA* IVS7 2498 is a polymorphism located in intron 7 of *PPARA*. The functional significance of this polymorphism has remained elusive but significant clinical associations have been found with this polymorphism. In LOCAT, *PPARA* IVS7 2498 (designated *“PPARA* intron 7 G/C polymorphism” in the publication) C allele carriers had a significantly greater progression of coronary atherosclerosis compared with GG homozygotes [50]. No pharmacogenetic interaction was noted [50]. When the association of *PPARA* IVS7 2498 polymorphism with coronary atherosclerosis was investigated in 3,012 healthy middle-aged men in NPHS2, *PPARA* IVS7 2498 CC homozygotes showed a trend toward greater incidence of ischemic events (myocardial infarction (MI) or coronary revascularization) (HR 1.83; 95% CI 0.96–3.51; *P* = .07) compared to *PPARA* IVS7 2498 CG heterozygotes and *PPARA* IVS7 2498 GG homozygotes [50].

#### 2.4.2. Left ventricular hypertrophy

The *PPARA* IVS7 2498 (designated *“PPARA* intron 7 G/C polymorphism” in the publication) has also been associated with physiologic left ventricular hypertrophy in 144 young male British army recruits undergoing a rigorous ten-week exercise program (mixed upper and lower body strength and endurance training) [67]. This polymorphism has also been associated with pathologic left ventricular hypertrophy in 1148 hypertensive men and women enrolled in an echocardiography substudy of the third monitoring trends and determinants in cardiovascular disease (MONICA) Augsburg study [67]. In both studies, the *PPARA* IVS7 2498 C allele was significantly associated with increased LV mass index [67].

### 2.5. PPARA IVS7 2498 G > C pharmacogenetic associations

#### 2.5.1. Response to fenofibrate

The Diabetes Atherosclerosis Intervention Study (DAIS) was designed to investigate if fenofibrate treatment of relatively mild dyslipidemia in 418 patients with type 2 DM would be associated with less progression of coronary atherosclerosis after treatment for at least 3 years with fenofibrate [68]. DAIS found that fenofibrate reduced the progression of angiographic coronary artery disease [69], the progression of microalbuminuria (an early marker of diabetic nephropathy, and an independent risk factor for cardiovascular disease) [70]; and although not powered to examine clinical events, there were fewer in the fenofibrate group compared to the placebo group [69]. Given these findings, the association between genetic variation in the *PPARA* IVS7 2498 polymorphism (designated “*PPARA* intron 7 G/C polymorphism” in the publication) and response to fenofibrate in DAIS was investigated [71]. DAIS subjects were divided into high responders (greater than 30% reduction, chosen because 30% was the mean reduction in DAIS) and low responders (less than 30% reduction) in their plasma triglyceride levels and the prevalence of *PPARA* IVS7 2498 genotype in the two groups was assessed [71]. Of the 85 high responders (55% of population), there was a significantly different prevalence of *PPARA* IVS7 2498 GG homozygotes (84.7%) when compared to the low responders (68.6%; *P* < .05) [71]. In stepwise logistic regression analysis, the best independent predictors of response to fenofibrate treatment were baseline triglyceride level and *PPARA* IVS7 2498 genotype (*PPARA* IVS7 2498 GG versus C allele carriers response to fenofibrate: OR 3.1; 95% CI 1.28–7.52; *P* = .012) [71].

#### 2.5.2. Response to acarbose

Investigators from the STOP-NIDDM trial were interested in whether *PPARA* polymorphisms would be associated with the conversion to type 2 DM in response to acarbose in patients with impaired glucose tolerance [72, 73]. They investigated this association with 11 SNPs located from exon 1 to exon 8 of *PPARA* and found that in the acarbose-treated group, *PPARA* IVS7 2498 (designated “rs4253778” in the publication) CC homozygotes had a 2.7-fold risk of developing type 2 DM (95% CI 1.14–6.79; *P* = .03) [74]. *PPARA* IVS7 1343 (designated “rs4253776” in the publication), a SNP located1,155 nucleotides upstream of *PPARA* IVS7 2498 and in moderate LD with *PPARA* IVS7 2498 (r^2^ of 0.565 in this population), also had an association with the development of type 2 DM [74]. *PPARA* IVS7 1343 G allele carriers had a 1.7-fold increased risk of developing type 2 DM (95% CI 1.04–2.88; *P* = .04) and a significant treatment by genotype interaction was observed [74].

## 3. PPARG

### 3.1. PPARG Pro12Ala genetic associations

#### 3.1.1. Metabolic traits and the development of type 2 DM

The *PPARG* Pro12Ala polymorphism is in exon B of *PPARG* which is specific to PPAR*γ*2, the PPAR*γ* isoform restricted to adipose tissue [75]. In vitro experiments have demonstrated that, compared to the *PPARG* Pro12 variant, the *PPARG* Ala12 variant has lower binding affinity for a PPAR responsive element and decreased PPAR*γ*-activation of a reporter construct in response to ligand [75]. The *PPARG* Pro12Ala polymorphism has been the most investigated PPAR polymorphism.

The association of the *PPARG* Pro12Ala polymorphism with metabolic traits and the risk/development of DM has been investigated in individuals of all ages and of different ethnicities including Chinese and Japanese individuals in the Hypertension and Insulin Resistance (SAPPHIRe) study, [76], Iranian individuals [77], obese Italian children, [78] middle-aged and elderly Finns, [75] and Spanish women [79]. Although most of these studies (including the ones mentioned here) report that *PPARG* Ala12 allele carriers have increased insulin sensitivity compared to *PPARG* Pro12 homozygotes, a recent meta-analysis of 57 studies reported that this association only held for certain subgroups [80]. When *PPARG* Ala12 allele carriers were compared to *PPARG* Pro12 homozygotes, only the obese subgroup demonstrated increased insulin sensitivity [80]. However, when *PPARG* Ala12 homozygotes were compared to *PPARG* Pro12 homozygotes (full genotype information that allowed this analysis was only available in 12 of the 57 studies), the association of the *PPARG* Ala12 allele with increased insulin sensitivity was more evident in all groups [80].

More recently, the association of the *PPARG* Pro12Ala polymorphism with metabolic traits and the risk of developing hyperglycemia over 6 years was investigated in 3,914 French Caucasians in the Data From an Epidemiological Study on the Insulin Resistance Syndrome (DESIR) cohort (of note, this study was not included in the meta-analysis as it was published after the meta-analysis was submitted) [81]. At baseline, *PPARG* Ala12 allele carriers had significantly lower fasting insulin and insulin resistance as determined by homeostasis model assessment of insulin resistance (*P* = .001, compared to *PPARG* Pro12 homozygotes) [81]. After 6 years of follow up, *PPARG* Ala12 allele carriers had significantly less increase in fasting insulin (*P* = .007, compared to *PPARG* Pro12 homozygotes) and insulin resistance (*P* = .018, compared to *PPARG* Pro12 homozygotes) [81]. In addition, after 6 years of follow up, *PPARG* Ala12 allele carriers who were normoglycemic at baseline (*n* = 3,498) had significantly less hyperglycemia, compared to compared to *PPARG* Pro12 homozygotes [81].

This data, as well as very recent data from 3,548 individuals in the diabetes prevention program (DPP) [82] confirmed two earlier meta-analyses (of the literature available at time of each meta-analysis publication) [83, 84]. This large study reported that *PPARG* Pro12 homozygotes had a 1.2-fold increased risk of developing type 2 DM (95% CI 0.99–1.57; *P* = .07) compared to *PPARG* Ala12 allele carriers [85]. This relative risk matched the 1.2-fold risk found in both meta-analyses (*P* = .002 in the meta-analysis performed by Altshuler et al.) [83, 84].

#### 3.1.2. Coronary and carotid atherosclerosis

Several studies have investigated the association of the *PPARG* Pro12Ala polymorphism with coronary artery disease and/or myocardial ischemic events, however, some have yielded contradictory results [86–88]. 14,916 men enrolled in the Physicians' Health Study [89] were followed for a mean of 13.2 years and the association between *PPARG* Pro12Ala polymorphism and MI was assessed [88]. *PPARG* Pro12Ala genotype was compared in 523 individuals who developed an MI, and 2,092 who did not show evidence of an MI [88]. Of those individuals who developed an MI, the frequency of *PPARG* Ala12 allele carriers was significantly less than in the controls, with a decreased risk of subsequent MI (hazard ratio HR = 0.77; 95% CI 0.60–0.98; *P* = .034) [88].This relationship held even after controlling for traditional cardiac risk factors.

In contrast, a study of 2,016 patients with type 2 DM from the genetic portion of the continually updated dataset known as the Diabetes Audit and Research in Tayside Scotland database (Go-DARTS) [87], a borderline, nonsignificant association of the *PPARG* Ala12 allele carriers with nonfatal MI or revascularization (HR 0.54; 95%CI 0.27–1.08; *P* = 0.08, compared to *PPARG* Pro12 homozygotes) was observed for the entire group. Subgroup analysis demonstrated a significant association if patients younger than 70 years old at time of enrollment were assessed separately (HR 0.43; CI 0.18–0.99; *P* = .05) or if patients younger than 70 year old at time of enrollment with no prior history of stroke, MI, or revascularization were evaluated for time to first event (HR 0.21; CI 0.06–0.69; *P* = .01) [87].

When the association of *PPARG* Pro12Ala polymorphism with the risk of coronary artery disease was assessed prospectively in women enrolled in the Nurses' Health Study (8 years mean follow up) and in men (6 years mean follow up) enrolled in the Health Professionals Follow-Up Study (HPSF) [86], carriers of the *PPARG* Ala12 allele again had an increased risk of MI [86]. 249 women and 266 men with MI were compared to nested case-controls and matched for age, smoking status, and phlebotomy date [86]. Men carriers of the *PPARG* Ala12 allele had an increased risk of MI or cardiac death (RR = 1.44; CI 1.00–2.07; *P* = .05) [86]. There was no statistical difference in nonfatal MI or cardiac death in women carriers of the *PPARG* Ala12 allele (RR = 1.17; CI 0.82–1.68; *P* = .39) [86]. When data were pooled for men and women, carriers of the *PPARG* Ala12 allele had an increase risk of MI or cardiac death (RR = 1.30; CI 1.00–1.67; *P* = .05) and, when stratified by body weight, men and women with a body mass index ≥25 kg/m^2^had a 1.68-fold increase in risk (CI 1.13–2.50; *P* = .01) [86].

A study of 267 Korean individuals (158 males and 109 females) referred for coronary angiography for chest pain, found no significant association between the *PPARG* Pro12Ala polymorphism and prevalence or severity of coronary artery disease [90]. While the results from these studies may seem contradictory, there are obvious differences in study design, patient cohorts, primary end-points, and power. In addition, it is possible that geographic and ethnic differences in allele frequencies may contribute to variability in the study findings.

An association has also been observed between the *PPARG* Pro12Ala polymorphism and carotid intima media thickness [91, 92]. In two studies involving over 300 patients, carriers of the *PPARG* Ala12 allele had less carotid intima media thickness measured by B-mode ultrasound [91, 92].

### 3.2. PPARG Pro12Ala pharmacogenetic associations

#### 3.2.1. Response to rosiglitazone

The *PPARG* Pro12Ala polymorphism resides in the ligand binding domain of PPAR*γ* and could therefore result in different affinity to bind TZDs. Variation in the *PPARG* Pro12Ala polymorphism and response to rosiglitazone was investigated in 198 men and women with type 2 DM (HbA_1C_ values between 7.5–11.5% and fasting glucoses between 140–250 mg/dL) treated with rosiglitazone for 12 weeks [93]. The decrease in fasting glucose in response to the drug was significantly greater in carriers of the *PPARG* Ala12 allele compared to *PPARG* Pro12 homozygous patients [93]. Improvement in HbA_1C_ was also significantly better in carriers of the *PPARG* Ala12 allele compared to *PPARG* Pro12 homozygous patients [93]. In addition, 86.67% of *PPARG* Ala12 allele carriers responded to rosiglitazone (defined by a greater than 15% decrease in HbA_1C_ levels and/or a greater than 20% decrease in fasting glucose level) compared to 43.72% of *PPARG* Pro12 homozygous patients (*P* = .002) [93].

#### 3.2.2. Response to acarbose

Investigators from the STOP-NIDDM trial were interested in whether PPAR polymorphisms would be associated with the conversion to type 2 DM in response to acarbose in patients with impaired glucose tolerance [72, 73]. They found that women treated with acarbose homozygous for the *PPARG* Pro12 allele had increased risk of developing type 2 DM compared to *PPARG* Ala12 allele carriers treated with acarbose (OR 2.89; 95% CI 1.20–6.96; *P* = .018) but found no significant difference in the men [72]. The authors did not provide an explanation for the gender differences.

### 3.3. PPARG 54,347 C > T genetic associations

#### 3.3.1. Coronary atherosclerosis

The *PPARG* 54,347 C > T polymorphism (also referred to as *PPARG* 161 C > T and *PPARG* 14,311 C > T) is a silent C > T substitution (i.e., does not cause an amino acid change in the protein) in nucleotide 161 of exon 6 [94]. No functional information on this polymorphism is available to date. The *PPARG* 54,347 C > T polymorphism has been associated with the extent of coronary artery disease by angiography [95], carotid intima media thickness [92], and incidence of MI among individuals younger than age 50 [96].

### 3.4. PPARG 54,347 C > T pharmacogenetic associations

#### 3.4.1. Response to fluvastatin

The Lipoprotein and Coronary Atherosclerosis Study (LCAS) was a randomized, placebo-controlled study of 429 subjects, 35–70 years old, with at least one 30–75% diameter stenosis on coronary angiography and LDL cholesterol of 115–190 mg/dL designed to assess the regression in coronary atherosclerosis (as measured by within-patient perlesion change in minimal lumen diameter by quantitative coronary angiography) in response to fluvastatin [97, 98]. After 2.5 years of treatment with fluvastatin, mean LDL cholesterol was reduced by 23.9%, and change in minimal lumen diameter by quantitative coronary angiography was significantly less in the fluvastatin-treated group (0.028 mm decrease in diameter in the fluvastatin-treated group compared to 0.100 mm decrease in diameter in the placebo group; *P* < .01) [99]. Clinical event rates had a trend towards benefit in the fluvastatin-treated group but were not statistically significant [99].

Genetic variation of *PPARG* 54,347 C > T (designated “*PPARG* 161 C > T” in the publication), *PPARG* Pro12Ala, and *PPARG* 25,506 C > T as well as the association with baseline lipid parameters and response to fluvastatin was assessed in 372 individuals from LCAS [100]. *PPARG* haplotype was associated with the degree of coronary atherosclerosis (mean number of coronary lesions; *P* = .026) and changes in minimum lumen diameter (*P* = .022) in response to fluvastatin [100]. *PPARA* and *PPARD* polymorphisms were also assessed: no associations were found with *PPARA* genotype or haplotype; *PPARD* associations are discussed below [100].

### 3.5. PPARG haplotype pharmacogenetic associations

#### 3.5.1. Response to troglitazone

The Troglitazone in the Prevention of Diabetes (TRIPOD) study was a placebo-controlled trial designed to test if TZD therapy could prevent the development of type 2 DM in Hispanic women with previous gestational DM [101, 102]. In this trial, the incidence of type 2 DM was decreased by 55% in the troglitazone-treated group (coincident with improvement in insulin sensitivity) compared to placebo [102]. Interestingly, 8 months after discontinuation of treatment, there remained a statistically significant difference in the development of type 2 DM between those treated with troglitazone and placebo (2.3% versus 15%; *P* = .03) [102].

In TRIPOD, 30% of women were classified as nonresponders as they were in the lowest tertile of 3 month improvement in insulin sensitivity and did not gain any protection from development of type 2 DM [102]. Although there was no association of the common, functional *PPARG* Pro12Ala polymorphism with response to troglitazone [103], there was an individual association of eight other *PPARG* polymorphisms with troglitazone response [104]. In addition, three hapolotypes blocks were defined that were independently, or jointly, involved in mediating the response to troglitazone [104]. Specifically, individuals with the most common haplotype within a haplotype block starting in intron 1, containing the A2 promoter and ending within intron 2 (designated “Block 1” in the publication) had an odds ratio of 2.22 for nonresponse to troglitazone (*P* = .032), and the most common haplotype within a haplotype, located completely within intron 2 (designated “Block 2” in the publication), had an odds ratio of 4.18 for nonresponse (*P* = .012) [104]. In addition, the most common haplotype within a haplotype located in the 3′ untranslated region of *PPARG* (designated “Block 5” in the publication) had a borderline significant odds ratio of 0.51 for response (*P* = .049) [104].

## 4. PPARD

### 4.1. PPARD −87 T > C genetic associations

#### 4.1.1. Dyslipidemias

The *PPARD* −87 T > C (designated “*PPARD* 294 T > C” polymorphism in the publication) was one of four polymorphisms identified by direct sequencing of the 5′ untranslated region of *PPARD* in 20 unrelated healthy subjects [105]. This polymorphism is located 87 base pairs upstream of the translation start site and 294 base pairs downstream from the transcription start site. In vitro experiments have demonstrated functional differences of the two variants and have implicated the transcriptional corepressor SP1 in contributing to the differences [106].

When the association of the *PPARD* −87 T > C polymorphism with variation in plasma lipid levels was investigated in 543 healthy men (and validated in an independent cohort of 282 healthy men), *PPARD* −87 CC homozygotes had increased plasma LDL cholesterol compared to *PPARD* −87 TT homozygotes [106].

#### 4.1.2. Coronary atherosclerosis and cardiac events

Skogsberg et al. investigated whether the *PPARD* −87 T > C polymorphism (designated “*PPARD* 294 T > C” polymorphism in the publication) was associated with increased plasma-LDL cholesterol levels and/or increased risk of having cardiac events. In the West Of Scotland Coronary Prevention Study (WOSCOPS), a randomized, double-blind, placebo-controlled trial with the primary goal of investigating the effect of pravastatin in preventing cardiac events in patients with mild-to-moderate hypercholesterolemia (LDL cholesterol between 4.5 and 6.0 mmol/L) [107]. Although carriers of the *PPARD* −87 C allele had a significantly lower HDL cholesterol compared with the PPARD −87 TT homozygotes, there was no association of this polymorphism with cardiac events and no genotype-by-treatment interaction [107].

### 4.2. PPARD haplotype pharmacogenetic associations

#### 4.2.1. Response to fluvastatin

Genetic variation of *PPARD* −87 T > C (designated “*PPARD* 294 T > C” in the publication) and *PPARD* −4401 C > T as well as the association with baseline lipid parameters and response to fluvastatin was assessed in 372 individuals from LCAS [100]. *PPARD* haplotype was associated with the degree of coronary atherosclerosis (mean number of coronary lesions) and changes in triglyceride (*P* = .01) and apoC-III (*P* = .047) levels in response to fluvastatin [100].

#### 4.2.2. Response to acarbose

Genetic variation in six SNPs in *PPARD* in patients with impaired glucose tolerance and association with the conversion to type 2 DM in response to acarbose was investigated in the STOP-NIDDM trial [72, 73]. Women treated with acarbose carrying the C allele of *PPARD* −48,444 C > T (designated “rs6902123” in the publication) had increased risk of developing type 2 DM compared to TT homozygous women treated with acarbose (OR 2.70; 95% CI 1.44–5.30; adjusted *P* = .002) [73].

## 5. CONCLUSIONS

With their pleiotropic effects on lipid metabolism, glucose homeostasis, myocardial energetics, and responses to ischemia, as well as the considerable evidence linking genetic polymorphisms identified within the PPAR complex to common cardiovascular diseases, the PPAR family of transcription factors is central to the regulation of a number of key cellular pathways that impact on normal and pathologic cardiovascular physiology and thus represent very promising targets for further advances in pharmacologic intervention. Early pharmacogenetic investigations into the associations of a select, few of these polymorphisms with patient responses to drug therapy have yielded important clues to commonly observed variability in both response and outcomes. Given the central role of the PPARs in critical metabolic pathways, this experience points the way to a future where knowledge of relevant PPAR genotype might be utilized to guide more appropriately tailored and individualized therapy.

## Figures and Tables

**Table 1:** 

	SNP	rs number
*PPARA*	Leu162Val	rs1800206
	Val227Ala	rs1800234
	IVS7 2498	rs4253778
	IVS7 1343	rs4253776

*PPARG*	Pro12Ala	rs1801282
	25,506 C > T	rs2028759
	54,347 C > T	rs3856806

*PPARD*	−87 T > C	rs9658134
	−4,401 C > T	rs2038068
	−48,444 C > T	rs6902123
